# A phase III trial comparing an anionic phospholipid-based cream and aloe vera-based gel in the prevention of radiation dermatitis in pediatric patients

**DOI:** 10.1186/1748-717X-2-45

**Published:** 2007-12-19

**Authors:** Thomas E Merchant, Christina Bosley, Julie Smith, Pam Baratti, David Pritchard, Tina Davis, Chenghong Li, Xiaoping Xiong

**Affiliations:** 1 Department of Radiological Sciences, St. Jude Children's Research Hospital, Memphis, TN, USA; 2 Department of Biostatistics, St. Jude Children's Research Hospital, Memphis, TN, USA

## Abstract

**Purpose:**

Radiation dermatitis is a common side effect of radiation therapy (RT). In severe cases, RT must be interrupted until the skin heals, which can compromise treatment. The purpose of the study was to compare an anionic polar phospholipid (APP)-based cream and an aloe vera-based gel to determine their effectiveness in preventing and treating radiation dermatitis.

**Patients and methods:**

Forty-five pediatric patients (median age, 11 years) with various diagnoses who received at least 23.4 Gy participated. APP cream and aloe vera gel were symmetrically applied within the irradiated field after each treatment. Three measures were collected before, during and after completion of treatment: subject's skin comfort, dermatologic assessment, and common toxicity criteria (CTC).

**Results:**

Significant differences in specific variables favoring APP cream use were noted in some patients including skin comfort variables, dry (*p *= 0.002), soft (*p *= 0.057), feels good (*p *= 0.002), rough (*p *= 0.065), smooth (*p = 0.012*) and dermatologic variables, dryness (*p *= 0.013), erythema (*p *= 0.002) and peely (*p *= 0.008). Grouped CTC scores were supportive of APP cream (*p *= 0.004). In comparing the first and last assessments, two dermatologic variables, dryness (*p *= 0.035) and peely (*p *= 0.016), favored APP cream.

**Conclusion:**

APP cream is more effective than aloe vera-based gel for prevention and treatment of radiation dermatitis.

## Background

The prevention and treatment of radiation dermatitis is required for all radiation oncology patients, regardless of the intensity of therapy. Skin care is an important function of the radiation oncology nursing staff, and the skin is routinely evaluated by the attending physician. Reducing skin toxicity is important, because it allows a patient to complete a continuous course of RT and minimizes the intensity of radiochemotherapy interactions that are common among patients who receive combined modality therapy. No product has been identified as the superior treatment for radiation dermatitis. Therefore, skin reactions remain a common cause of patient discomfort and cancer treatment delay.

The epidermis (the outer skin) consists of four layers: the stratum basale (the internal layer), the stratum spinosum, the stratum granulosum, and the stratum corneum (the surface layer). The stratum corneum is impermeable, and its cells, the corneocytes, are considered dead tissue. The sebaceous glands secrete sebum (oil) onto the impermeable surface of the skin. Sebum is mostly triglyceride in character and chemistry, and it provides an occlusive oil film barrier on the surface of the skin to regulate evaporation of water. Strategies aimed at protecting the skin from desiccation and degradation focus on the lamellar structure of the stratum cornea and maintenance of the lipid bilayers, which requires a combination of external and internal oils and moisture (hydration) [1]. Phospholipids are key molecules in the formulation of products that maintain the lamellae, and contemporary skin care technology has made it possible to mix oil and water to create products that can be used to keep skin soft, smooth, and supple [2].

Proactive treatment to prevent radiation dermatitis is directed at reducing the drying effect of radiation on the skin and involves instructing patients to avoid irritating the irradiated region. Dryness due to RT leads to desquamation and loss of the superficial protective layers of the skin including lipid barriers. Simple moisturizers are applied in an effort to hydrate the skin and form a barrier to transcutaneous water loss and topical steroids are applied to reduce pruritus.

To date, there is no consensus regarding the optimal management of radiation dermatitis [3-5], and treatment often follows the management of other dermatoses. There are a number of reports from prospective controlled clinical studies for breast cancer patients including phase II and III trials comparing different agents in the treatment of radiation dermatitis. In one Phase III trial, 194 female patients receiving breast or chest-wall irradiation were randomized to receive an aloe vera gel or placebo gel, and 108 female patients undergoing the same treatment received either aloe vera gel or no treatment. The investigators concluded that aloe vera gel did not protect against RT-induced dermatitis [6]. One study concluded that biafine cream (water-based emulsion) was useful to avoid delays or interruptions after chemo-radiotherapy for breast cancer even though the majority of patients developed Grade 2 radiation dermatitis[7]. Other studies in similar patient populations have not shown that Biafine and Lipiderm have a radioprotective effect on the skin [8] nor that Biafine is better than best supportive care [9]. Agents incorporating hyaluronic-acid [10], potent topical corticosteroids [11], or specific plant extracts (calendula) [12] have shown promising results in the prevention and treatment of acute dermatitis though suppression of cytokine responses and inflammation or immune cell modulation. In another trials, hydrogel or dry dressings [13], and sucralfate or aqueous creams [14] have been tested on their ability to reduce the time to healing of moist desquamation after radiotherapy to the head-and-neck, breast, or anorectal areas.

We used a novel anionic polar phospholipid (APP)-based skin cream in a side-by-side comparison in individual patients in the same manner that we routinely perform prophylactic skin care. The APP cream was previously evaluated in a double-blind trial for the prevention and control of dryness, inflammation, and fissures on the feet of patients with diabetes [15]. Similar agents have been considered to replenish the tear film phospholipid layer [16]. The purpose of this study was to compare the effectiveness of the novel APP cream with that of aloe vera gel in the prevention of radiation dermatitis in children treated with fractionated external-beam irradiation. Variables tested included the subjective assessments by the patient of skin dryness, softness, satisfaction, roughness, and smoothness and examiner assessment of skin dryness, erythema, and peeling.

## Methods

This study was approved by the St. Jude Institutional Review Board, and informed consent was obtained from the patient or guardian, as appropriate, before the patient was enrolled. Study criteria included age older than 3 years and younger than 21, a diagnosis that required external-beam irradiation, no prior history of RT at the site to be evaluated, a prescribed total dose of RT greater than or equal to 23.4 Gy, no anticipated use of superficial tissue compensators ("bolus"), no pre-existing dermatologic condition that would preclude the evaluation of the skin at the site to be treated (infection, trauma, collagen vascular disease), no contraindications to the use of the study treatments or any of their components, and adequate performance status as determined by the ECOG (Eastern Cooperative Oncology Group) scale (0–3) [17].

### Evaluations, tests, and observations

There were three observation measures: (1) the subject skin comfort assessment, (2) clinical dermatologic assessment, and (3) assessment by the Common Toxicity Criteria (CTC) Version 1.0 of the National Cancer Institute. These measures were obtained before initiation of RT, weekly during treatment, and at the time of first follow-up examination, which typically occurred 4 to 6 weeks after completion of RT. These measures were also obtained at the time of skin care failure. Each patient underwent two to six evaluations that included all three measures.

**Figure 1:**
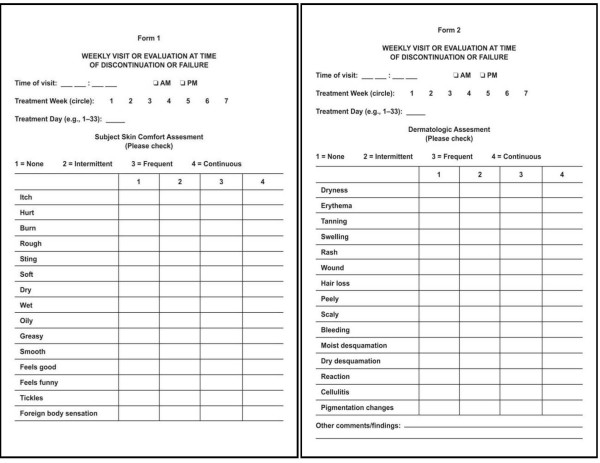
Study Questionnaires. The Subject Skin Condition Assessment form (A) and Dermatologic Assessment form (B) are presented.

The subject skin comfort assessment was completed by the patient or a parent. The assessment consisted of 15 items (variables), each on a 4-level scale (Figure 1). This assessment included both positive items (e.g., "soft" and "feels good") and negative items (e.g., "itch" and "dry"). The dermatologic assessment (Figure 1), which was completed by the nursing staff, was a similar questionnaire but consisted of negative items only (e.g., "dryness" and "erythema"). The CTC for adverse events involving the skin was as follows: grade 1 – none or no change; grade 2 – scattered macular or papular eruption or erythema that is asymptomatic; grade 3 – scattered macular or papular eruption or erythema with pruritis or other associated symptoms; grade 4-generalized symptomatic macular, popular, or vesicular eruption; grade 5 – exfoliative dermatitis or ulcerating dermatitis.

### Pretreatment evaluation

**Figure 2:**
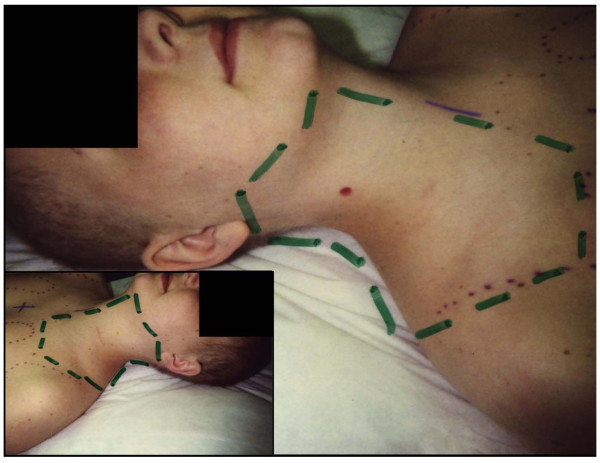
Photograph of patient who received mantle irradiation and outline of symmetric areas for study.

Patients underwent fluoroscopic simulation before actual therapy was initiated. After the simulation, the patient was evaluated in the radiation oncology clinic by the attending physician and nursing staff. Study questionnaires were completed once the anatomic study region (*region, site*, and *area *are used interchangeably) was defined, divided into two parts, and photographed (Figure 2). The study focused on patients whose radiation treatment fields allowed for easy access and examination. Patients who received craniospinal irradiation or mantle irradiation had symmetrical irradiation of the region between the mastoids and the clavicles; these regions were often chosen for ease of study. Patients who received RT to an extremity, the trunk, or abdomen were included if the homogeneity of radiation dose permitted a well-defined anatomic region to be evaluated.

### Treatment and evaluation during radiation therapy

Patients received at a minimum conventionally fractionated doses of 1.5–1.8 Gy, delivered once a day. The minimum total dose was 23.4 Gy. Each day, after completing RT, the patient was seen in the radiation oncology clinic by a nurse who applied the aloe vera gel and the APP skin cream to the designated study site. The treating radiation oncologist evaluated the patient once during each interval of five treatments. At that time, a dermatologic exam was performed, and study questionnaires were completed. Each evaluation was designated according to the treatment day (day 5, day 10, day 15, etc.); the patients were also evaluated on the last day of treatment, which often did not coincide with a weekly evaluation.

### Identification of skin care failure

Skin care failure was identified by one of two means: Either patients informed the nursing staff that their skin was dry to the extent that the resultant pruritus was unbearable, or the nursing staff noticed a transition from dry to moist desquamation. Dermatologic examination and subject skin comfort assessment questionnaires were performed after skin care failure; the next level of skin care was administered to the site in which that specific product had failed; and no further data were gathered for the failed site. Both sites were continually evaluated on a weekly basis. According to the standards of practice at the time, follow-up examinations were done 4 to 6 weeks after completion of RT.

### Treatment and evaluation after Radiation Therapy

Patients returned to the radiation oncology clinic 4 to 6 weeks after completion of RT for routine follow-up. During that visit, questionnaires were completed, and photographs were taken of the treatment site if skin care failure had not occurred at both sites. The evaluation was similar to that given during RT.

### APP skin cream

The APP skin cream (Ocular Research of Boston (ORB), Inc, Boston, MA) is a novel oil-in-water emulsion that was prepared in an FDA-approved facility under cGMP guidelines, but it is not commercially available. The active ingredients of APP cream are triglycerides and phospholipids preserved with benzyl alcohol, methyl paraben, propyl paraben, and diaxolipinyl urea. It was applied topically and liberally to the affected area with the bare hand. Application of the cream was accomplished with the ventral surface of the fingers using a rotary motion of the fingers with light pressure to the skin. The cream was massaged into the skin until the surface of the skin no longer felt greasy. Inadequate application was noted by the appearance of a white residual film on the skin.

### Aloe vera gel

The aloe vera gel which was commercially available, contained water, aloe vera, D-panthenol, triethanolamine, carbomer 934P, hyaluronic acid, potassium sorbate, diazolidinyl urea, methylparaben, and propylparaben. The gel was applied in a manner identical to that described above for the APP cream.

### Statistical considerations

The study was designed as a prospective and randomized Phase III clinical trial with a planned accrual of 45 eligible patients. The APP cream and aloe vera gel were symmetrically and adjacently applied to the irradiated sites in individual patients; the side treated with cream or gel was chosen randomly for each patient at the beginning of treatment, and this status was kept for the entire process of RT. The primary endpoint is the skin care failure which included onset of moderate-to-severe dryness, pruritus, erythema, and dry desquamation. For each individual patient, the cream is better than gel if gel fails before cream does; and vice versa. The sample size for the study was calculated to test whether the cream is better than the gel, or equivalently to test the hypothesis that the proportion of patients for whom the cream is better than gel is more than 50%. 45 patients were planned for this study to detect a proportion of 70% (cream better than gel) with a power of 0.86 and a significance level of 0.05.

At pre-treatment, no statistical test was performed because the scores of both subject skin comfort and dermatologic assessments, along with CTC scores, were identical between cream and gel in all 45 patients. During radiation therapy we compared the probability that cream is better than or equal to gel with the probability that gel is better than or equal to cream using a conditional test with binomial distribution. Longitudinal mixed models were not considered appropriate for assessment score comparison during radiation therapy because cream and gel had identical scores in a number of patients. At follow-up, t-tests were performed to compare scores of cream and gel.

Statistical analyses were performed using either SAS^® ^(Cary, North Carolina) or StatExact-5 (Cytel Software Corporation, Cambridge, Massachusetts). The statisticians were blinded as to knowledge of cream or gel during the analysis. The significance level was at type I error rate alpha = 0.05 for all tests. The P-values were not adjusted for multiple testing.

## Results

The trial included 45 pediatric patients whose average age was 10 years (range, 3–19 years). The average total dose of radiation was 34.3 Gy (range, 25.2–67 Gy). The most common diagnoses were Hodgkin disease (n = 16), CNS tumor (n = 10), pediatric sarcoma (n = 8), and neuroblastoma (n = 6). The most common treatment sites were the thorax, upper thorax, axilla, and craniocervical regions. One patient with two sites to assess was removed from the final analysis because they were the lone case with two sites.

### Pretreatment assessment

Before RT was initiated, we assessed the effects of the APP cream and aloe vera gel on each patient's skin within the planned field of irradiation. We found no difference between the two products in either the score distributions for the subject skin comfort assessment or the dermatologic assessment.

### Assessment at follow-up

The analysis at follow-up was performed to detect longer term differences between the two agents. Data for 29 patients (64%) were available at follow-up (7 ± 7 weeks, median ± SD). Because both products were applied to the skin of each patient, a paired *t*-test was used for this analysis. Patients gave the same or similar scores to the APP cream and aloe vera gel for 11 of 15 (73%) variables: hurt, burn, sting, wet, oily, greasy, feels good, feels funny, tickles, foreign body sensation. Of the remaining variables, the score for "softness" tended to favor the use of the APP cream (*p *= 0.083; Table 1). Because the scores for the two products were identical before RT, the difference in scores at follow-up reflects a difference in long-term efficacy. At follow-up, the scores for the dermatologic assessment and common toxicity criteria were the same for both products.

**Table 1:** Follow-up assessment of Subject Skin Comfort after APP cream and aloe vera gel treatment to prevent radiation dermatitis.

**Variable**	Mean difference*	**SE**	***p*-value**
**Soft**	0.2069	0.1151	0.083
**Rough**	-0.069	0.0479	0.161
**Itch**	-0.034	0.0345	0.326
**Dry**	-0.034	0.0604	0.573
**Smooth**	0	0.0496	1.0

* The mean difference was calculated by *cream score – gel score *for each variable.

### Assessments during RT (weeks 1–6)

There were four possible outcomes comparing APP cream with aloe vera gel: (1) no difference; (2) cream ≥ gel, in which the patients' APP cream score was never less than their aloe vera gel score; (3) gel ≥ cream, in which the patients' aloe vera gel score was never less than their APP cream score; and (4) alternating, in which the product with the higher score varied throughout the trial.

Most patients had the same scores on many assessment variables. The *gel ≥ cream *outcome was found more often than the *cream ≥ gel *outcome on the negative variables (i.e., Dry, Rough, Itch, Dryness, Erythema, and Peely) (Table 2). This finding suggests that although there was no difference between the two agents in most patients; in some patients the APP cream was better than the aloe vera gel.

**Table 2:** Comparison of APP cream and aloe vera gel effectiveness during RT.

**Assessment**	Variable*	**No difference**	**Cream > Gel**	**Gel > Cream**	**Cream > Gel, Gel > Cream**	***P*-value**^†^
Subject Skin Comfort	Dry	25	2	15	2	0.002
	Soft	27	11	3	3	0.057
	Feels good	28	13	1	2	0.002
	Rough	31	2	9	2	0.065
	Smooth	32	10	1	1	0.012
Dermatologic	Dryness	25	3	14	2	0.013
	Erythema	33	0	10	1	0.002
	Peely	35	0	8	1	0.008
Common Toxicity Criteria	CTC value	34	0	9	1	0.004

*The p-values of all variables listed, except CTC value, were less than 0.05.

^†^Two-sided exact *p*-value for equality of *cream > gel *frequency and *gel > cream *frequency.

During RT, there was a difference in the CTC score favoring the cream (P = 0.004). The cream CTC score in all patients during RT was no larger than the gel CTC score. On the other hand, in 9 patients (20%) the gel CTC score was larger than cream CTC score at some point during RT.

### Comparing the first and last assessments

Because both agents were administered in the same manner and on the skin of the same patient, we removed the individual effects by analyzing the difference of the two scores (i.e., *gel score *– *cream score*) rather than the raw data. We also compared the difference at the first assessment with that at the last assessment. There were three possible outcome groups: (1) *first = last *group, this group included those whose scores showed no change in *gel – cream *over time; (2) *first < last *group, in which the *gel – cream *difference increased during the trial; and (3) *first > last *group, in which the *gel – cream *difference decreased during the trial.

The frequencies of the outcome groups are given for statistically significant variables in Table 3.

**Table 3:** Comparison of the First and Last Assessments of APP Cream and Aloe Vera Gel Effectiveness in Preventing Radiation Dermatitis in Pediatric Patients

** Assessment**	**Variable**	First = Last*	***First < Last***	***First > Last***	*P*-value**
Subject Skin Comfort	Smooth	36	1	7	0.070
Dermatologic	Dryness	29	12	3	0.035
	Peely	37	7	0	0.016
	Scaly	39	5	0	0.063

* The difference was calculated by *cream score – gel score at first and last assessments *for each variable.

**Two-sided exact P-values

### Treatment failure

Treatment failure according to the definitions of the study occurred in 3 patients. Two patients had rhabdomyosarcoma and one desmoids tumor with doses ranging from 45–50.4 Gy. The two patients with rhabdomyosarcoma received concurrent chemotherapy. Failure occurred in cream and gel treated sites simultaneously in two and in the cream treated site prior to the gel treated site in one patient.

## Discussion

Prior to this trial and after radiation therapy was initiated, the skin was usually treated daily with an aloe vera-based gel. The patients or their caregivers would apply the gel lightly and attempt to avoid any skin markings that facilitate localization of daily RT. When the prophylactic treatment failed, as determined by the onset of moderate-to-severe dryness, pruritus, erythema, and moist desquamation, the next level of care was then instituted. This generally included a cleansing spray and moisture barrier that contained zinc. If the patient experienced progressive moist desquamation, peeling, or itching that was not relieved with the cleansing spray or moisture barrier, then a third level of care was indicated. This level included a cleansing spray with the addition of alternating hydrocortisone-containing cream (1%) and hydrocortisone (1%)/Clioquinol (3%) cream.

The study demonstrated the superiority of a phospholipid-based cream over an aloe vera based gel in the prevention of radiation dermatitis in children receiving more than 23.4 Gy. This conclusion is based on a statistical analysis of subject skin comfort and dermatologic assessment performed before, during, and after RT. The APP cream was favored during treatment for the subject comfort variables of dry (0.002), softness (p = 0.057), feels good (p = 0.002), and smoothness (p = 0.012). The APP cream was also more efficacious during treatment for the dermatologic variables of dryness (0.013), erythema (p = 0.002), and peely (p = 0.008). The similar subject skin comfort assessment, dermatologic assessment, and common toxicity criteria scores observed when the first and last treatments were compared, as well as those from pretreatment and follow-up assessments was surprising given that 36 of 45 patients were treated with chemotherapy before or during RT. Possible explanations would include the relatively low dose of RT required for eligibility and the rigor of the trial in applying skin care products on a daily basis by trained personnel. This is further supported by our results where failure occurred in only 3 patients, two of whom received concurrent chemotherapy. One might expect that a greater proportion of patients would show a change over time, regardless of the agent applied, if the total radiation dose was higher. For the design of future trials, one might restrict the study cohort to include patients who have the same diagnosis, RT site, and cancer treatment regimen. Also, assessment of patients who received a higher total dose of radiation may be informative.

Cytokine and cellular responses to radiation therapy in the skin have been investigated to identify targets to mitigate the consequences of ionizing radiation therapy [18]. Commercial skin creams are primarily used to maintain the outer surface of the stratum corneum. These creams address only symptoms (e.g., dryness, flaking, or itching) and not the primary cause of most skin maladies: compromised bilayers and lamellae. The extracellular space between the corneocytes is filled with polar lipids that form the bilayers and lamellae (or polar membrane bilayers).

Each lipid bilayer of the lamellae system is separated from adjacent lipid bilayers by a water layer. These stacked lamellae fill the space among the corneocytes. If the lipids are compromised, then a wide range of skin disorders may result, including dryness, flaking, cracking, and accelerated aging. However, the organization of the lamellae is a biochemical process; therefore, lamellae can repair themselves without intervention by living cells.

Desiccation of the lamellar system causes the bilayers to align themselves into a crystalline-like structure that is hard and brittle, and rehydration will re-establish the organized bilayer system. Other factors degrade the skin and are not helped by the current generation of skin products.

Two mechanisms of action are unique to the APP cream: the repair of the lamellar system via the penetration of APP and triglycerides into the bilayers of the stratum corneum and the organization of water through the charged nature of the molecules involved. This action repairs defects (holes) in the strata that result from skin damage and resultant loss of natural polar lipid components.

Two components of the cream formulation address lamellar defects: APP and the triglycerides. Both molecules are polar and water seeking; therefore, they are attracted to the water layers of the stratum corneum, other layers of the epidermis, and the underlying dermis, depending on how severely the skin layers have been damaged. The driving forces for this penetrating action are the thermodynamic forces involved: the amphiphilic interactions, hydrogen bonding with water, electrostatic interactions, and the hydrophobic interaction (the force that results in water organization).

When APP molecules penetrate the stratum corneum and arrive at a defective lipid bilayer, they insert themselves alongside other polar lipids in the existing bilayer. Local forces orient the APP molecules appropriately (i.e., hydrophilic ends to hydrophilic ends and hydrophobic ends to hydrophobic ends). Like a zipper closing, the repeated insertion of APP molecules fills the lamellar defect, seals the gap, and thus re-establishes the water barrier.

## Conclusion

Reducing radiation-related toxicity is a central objective in radiation oncology. The use of advanced methods of treatment planning and delivery serve as examples of the effort that is undertaken to diminish the toxicity of RT in the adult and pediatric patient populations. As our ability to reduce RT-related toxicity through technologic initiatives plateaus, the importance of protecting normal tissues will increase.

## Competing interests

The author(s) declare that they have no competing interests.

## Authors' contributions

TM conceived of the study, participated in the analyses and drafted the manuscript. CB, JS, PB and DP conducted patient exams and participated in data collection. TD participated in the collection of data and data analyses. CL and XX assisted in writing the manuscript. All authors read and approved the final manuscript.
